# Thyroid Parameters and Kidney Disorder in Type 2 Diabetes: Results from the METAL Study

**DOI:** 10.1155/2020/4798947

**Published:** 2020-03-28

**Authors:** Yi Chen, Wen Zhang, Ningjian Wang, Yuying Wang, Chiyu Wang, Heng Wan, Yingli Lu

**Affiliations:** Institute and Department of Endocrinology and Metabolism, Shanghai Ninth People's Hospital, Shanghai Jiao Tong University School of Medicine, Shanghai, China

## Abstract

**Objective:**

Diabetic kidney disease is one of the most common microvascular complications of diabetes mellitus. We aimed to analyze the association of thyroid parameters with kidney disorders, especially in euthyroid participants.

**Methods:**

The data were obtained from a cross-sectional study, the METAL study. Thyroid parameters, including thyroid-stimulating hormone (TSH), free triiodothyronine (FT_3_), free thyroxine (FT_4_), triiodothyronine (T_3_), thyroxin (T_4_), thyroid peroxidase antibody (TPOAb), and thyroglobulin antibody (TgAb), of 4136 participants with type 2 diabetes were measured. Two structure parameters of thyroid homeostasis, including the sum activity of step-up deiodinases (SPINA-G_D_) and thyroid secretory capacity (SPINA-G_T_), and two pituitary thyrotropic function indices, including Jostel's TSH index (TSHI) and the thyrotroph thyroid hormone resistance index (TTSI), were also calculated. Kidney disorders were described according to the presence of reduced estimated glomerular filtration rate (eGFR) and/or higher urinary albumin to creatinine ratio (UACR).

**Results:**

The prevalence of kidney disorders increased with decreasing FT_3_ or T_3_ and increasing FT_4_ or T_4_ quartile levels (all *P* < 0.05). After full adjustment, linear regression showed that UACR levels were negatively associated with FT_3_ and T_3_ (*P* < 0.001). In addition, eGFR was positively associated with FT_3_ and T_3_ and was negatively associated with TSH and FT_4_ levels and TgAb positivity (all *P* < 0.05). By using binary logistic regression, higher TSH and FT_4_ and lower FT_3_ and T_3_ were associated with kidney disorders (all *P* < 0.05). Similar results were seen in sensitivity analyses, which were performed in 3035 euthyroid diabetic participants; however, TSH was no longer related to them. The area under the receiver operating characteristic curve (AUROC) of lower FT_3_ for existing kidney disorder was greater than that for any other thyroid hormones (all *P* < 0.001). The cutoff value of FT_3_ for reduced eGFR was 4.39 pmol/L. Regarding thyroid homeostasis parameters, SPINA-G_D_ was negatively associated with three statuses of kidney disorders, and TSHI and TTSI were positively associated with reduced eGFR (all *P* < 0.05).

**Conclusions:**

Among patients with type 2 diabetes, elevated TSH and FT_4_ (or T_4_), lower FT_3_ (or T_3_), TgAb positivity, lower SPINA-G_D_, and higher TSHI and TTSI were associated with kidney disorders. The lower FT_3_, even within the normal range (<4.38 pmol/L), may be the factor most related to reduced eGFR compared with other thyroid hormones in diabetic patients.

## 1. Introduction

Diabetes mellitus (DM) and its complications have become highly prevalent and have gained increasing attention [1], especially in developing countries. The estimated prevalence of diabetes among a representative sample of Chinese adults was 11.6%, which indicates the importance of diabetes as a public health problem in China [2]. Chronic kidney disease (CKD) is defined by the sustained presence of reduced kidney function or damage, often resulting from diabetes mellitus (DM) and hypertension [3]. Approximately 13% of individuals in the general US population have CKD, and the incidence is increasing globally [4–6]. Diabetic kidney disease (DKD) is one of the most common microvascular complications of diabetes mellitus and the leading cause of end-stage renal disease (ESRD) worldwide [7]. Approximately 20%–40% of patients with diabetes progress to DKD, and 40% also progress to ESRD [8]. Reduced estimated glomerular filtration rate (eGFR) and higher urinary albumin to creatinine ratio (UACR) levels are two main markers of diabetic kidney status, quantifying renal function and serving as a proxy of renal damage severity, respectively [3, 9].

The thyroid gland is one of the most important organs in the human body. It regulates the majority of the body's physiological actions [10]. Thyroid hormone has an impact on renal tubular function and the renin-angiotensin system and is associated with hemodynamic and cardiovascular alterations that interfere with renal blood flow [11]. Conversely, the kidney is not only an organ for the metabolism and elimination of TH but also a target organ of some of the actions of the iodothyronines [12]. Acute kidney injury and chronic kidney disease are accompanied by notable effects on the hypothalamus-pituitary-thyroid axis. Hormonal derangements at the level of the hypothalamic-pituitary axis are often seen with the worsening of kidney function, and recent evidence points towards the implication of such hormonal disorders in the genesis of CKD [13]. Thyroid dysfunction causes remarkable changes in glomerular and tubular functions and in electrolyte and water homeostasis [12].

Although several studies have suggested the association of thyroid disorders and CKD or DKD with conflicting results [7, 14–16], studies focusing on the possible relationship between thyroid status and eGFR and UACR among a relative number of DM participants, especially in euthyroid DM patients, have been limited. The present cross-sectional study investigated whether thyroid parameter concentrations, including thyroid-stimulating hormone (TSH), free triiodothyronine (FT_3_), free thyroxine (FT_4_), T_3_, T_4_, thyroid peroxidase antibody (TPOAb), and thyroglobulin antibody (TgAb), and four parameters of thyroid homeostasis, including the sum activity of step-up deiodinases (SPINA-G_D_), thyroid secretory capacity (SPINA-G_T_), Jostel's TSH index (TSHI), and thyrotroph thyroid hormone resistance index (TTSI), were related to eGFR, UACR, and the prevalence of kidney disorders, described as reduced eGFR, higher UACR, and higher UACR and/or reduced eGFR in type 2 diabetes.

## 2. Methods

### 2.1. Study Design and Participants

**Figure 1:**
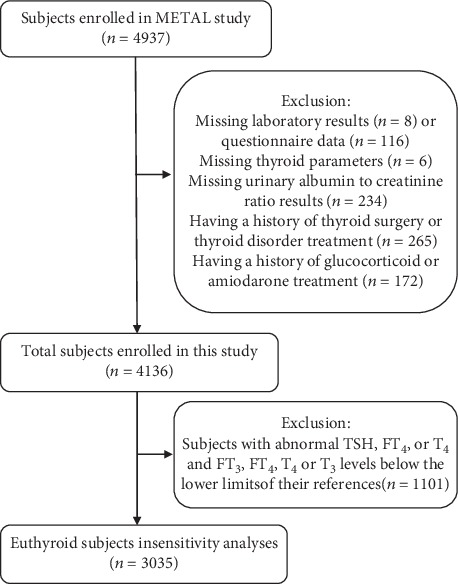
Flowchart of the inclusion and exclusion of participants.

The data of this study were obtained from a cross-sectional study, the METAL study (Environmental Pollutant Exposure and Metabolic Diseases in Shanghai, http://www.chictr.org.cn, ChiCTR1800017573) [17, 18], which was designed to investigate the association between exposure to heavy metals and diabetic complications in Chinese diabetic adults. We enrolled participants from seven communities in the Huangpu and Pudong districts in Shanghai. In 2018, we obtained the list of diabetic patients who were Chinese citizens ≥ 18 years old and had lived in their current area for ≥6 months from the registration platform in each community healthcare center and then randomly selected 50% of them (*n* = 4937) to receive the examination by using SPSS Statistics, Version 22 (IBM Corporation, Armonk, NY, USA). We excluded participants who were missing laboratory results (*n* = 8) or questionnaire data (*n* = 116), those who were missing thyroid parameters (*n* = 6) or UACR results (*n* = 234), those having a history of thyroid surgery or thyroid disorder treatment (*n* = 265), or those having a history of glucocorticoid or amiodarone treatment (*n* = 172) in this study. Finally, 4136 participants were involved in this study. Then, we excluded subjects with abnormal TSH, FT_4_, or T_4_ levels and those with FT_3_ or T_3_ levels below the lower limits of their reference ranges for sensitivity analysis (Figure 1). All participants provided written informed consent before data collection. The study protocol was approved by the Ethics Committee of Shanghai Ninth People's Hospital, Shanghai Jiao Tong University School of Medicine. All procedures followed were in accordance with the ethical standards of the responsible committee on human experimentation (institutional and national) and with the 1975 Declaration of Helsinki, as revised in 2008.

### 2.2. Laboratory Measurements

Serum samples for laboratorial assays were obtained by venipuncture after an 8-hour fast. Blood samples were stored at -20°C when collected and shipped by air in dry ice to one central laboratory, which was certified by the College of American Pathologists (CAP), within 2-4 hours of collection.

Serum TSH, FT_3_, FT_4_, T_3_, T_4_, TPOAb, and TgAb were measured by electrochemiluminescence (Roche, E601, Germany). The normal reference ranges for TSH, FT_3_, FT_4_, T_3_, T_4_, TPOAb, and TgAb were 0.27-4.20 mIU/L, 3.10-6.80 pmol/L, 12.00-22.00 pmol/L, 1.30-3.10 nmol/L, 66.00-181.00 nmol/L, 0-34.00 U/mL, and 0-115.00 U/mL, respectively. Euthyroid was defined as TSH, FT_4_, and T_4_ levels within normal limits and without FT_3_ or T_3_ levels below the lower limits. Two structure parameters of thyroid homeostasis, SPINA-G_D_ and SPINA-G_T_, as well as two pituitary thyrotropic function indices, TSHI and TTSI, were calculated according to previous studies [19–21].

Creatinine (Cr) and uric acid (UA) were measured by Beckman Coulter AU680 (Brea, USA). Using morning fasting spot urine samples, the concentrations of albumin and CR were measured with a Beckman Coulter AU680 (Brea, USA) using a turbidimetric immunoassay and an enzymatic method, respectively. Reduced eGFR was defined as eGFR < 60 mL/min/1.73m^2^ according to the Chronic Kidney Disease Epidemiology Collaboration (CKD-EPI) for “Asian origin” [22] and regardless of whether the UACR was high. Higher UACR was defined as UACR ≥ 30 mg/g, suggested by an ADA statement [23] and regardless of the value of eGFR. Higher UACR and/or reduced eGFR was defined as the positivity of higher UACR and/or reduced eGFR.

Lipid profiles, including triglycerides (TG), total cholesterol (TC), and high- (HDL-C) and low-density lipoprotein (LDL-C), were measured by Beckman Coulter AU680 (Brea, USA). Glycated hemoglobin (HbA1c) was assessed by high-performance liquid chromatography (MQ-2000PT, Medconn, Shanghai, China).

### 2.3. Clinical Parameters

A questionnaire about sociodemographic characteristics, medical history, family history, and lifestyle factors was adopted during the interview. The same group of trained and experienced personnel in the SPECT-China study (Survey on Prevalence in East China for Metabolic Diseases and Risk Factors, ChiCTR-ECS-14005052, http://www.chictr.org.cn) [24, 25] conducted the interviews and clinical examinations, including measurements of weight, height, and blood pressure, according to a standard protocol. Body mass index (BMI) was calculated as weight in kilograms divided by height in meters squared. Current smoking was defined as having smoked at least 100 cigarettes in one's lifetime and currently smoking cigarettes [2]. Hypertension was assessed by systolic blood pressure ≥ 140 mmHg, diastolic blood pressure ≥ 90 mmHg, or self-reported previous diagnosis of hypertension by physicians or medication use for hypertension. Dyslipidemia was defined as TC ≥ 6.22 mmol/L, TG ≥ 2.26 mmol/L, LDL‐C ≥ 4.14 mmol/L, HDL‐C < 1.04 mmol/L, self-reported previous diagnosis of hyperlipidemia by physicians, or medication use for dyslipidemia, according to the modified National Cholesterol Education Program-Adult Treatment Panel III.

### 2.4. Statistical Analysis

We performed survey analyses with IBM SPSS Statistics. All analyses were two-sided. A *P* value < 0.05 was taken to indicate a significant difference. Normally and nonnormally distributed continuous variables were expressed as mean ± SD and median (interquartile range), respectively. Categorical variables are presented as percentages. Pearson's *χ*^2^ test was used for dichotomous variables. Associations among thyroid parameters and two kidney parameters (UACR and eGFR) were analyzed using linear regression models with each measure as the outcome. The regression models were adjusted for age, sex, HbA1c, BMI, UA, the duration of diabetes, current smoking, dyslipidemia, hypertension, and the use of metformin. UACR and eGFR were ln-transformed because of their skewed distribution. The results were expressed as *B* values and 95% confidence intervals (CIs). The relationship of thyroid hormones and parameters of thyroid homeostasis with reduced eGFR, higher UACR, and higher UACR and/or reduced eGFR (all categorical variables) were assessed by logistic regression. The regression models were adjusted for age, sex, HbA1c, BMI, UA, the duration of diabetes, current smoking, dyslipidemia, hypertension, and the use of metformin. The results were expressed as odds ratios (ORs) (95% CIs). Area under the receiver operating characteristic curve (AUROC) analyses were used to compare the predictive powers of thyroid parameters for reduced eGFR, higher UACR, and higher UACR and/or reduced eGFR. Sensitivity analyses were performed in euthyroid participants with type 2 diabetes.

## 3. Results

### 3.1. Clinical Characteristics of the Participants

This study recruited 4136 participants with type 2 diabetes, with a mean age of 67.23 years old (SD 8.63, max 99, min 23); among them, 1983 were men. The clinical characteristics of all these participants are shown in Table 1. The mean duration of diabetes of the participants was 9 years, and the mean level of HbA1c was 7.2%. The prevalence of reduced eGFR, higher UACR, and higher UACR and/or reduced eGFR was 5.58%, 26.0%, and 28.1%, respectively.

### 3.2. Prevalence of Kidney Disorders according to Thyroid Hormone Quartile Levels

**Figure 2:**
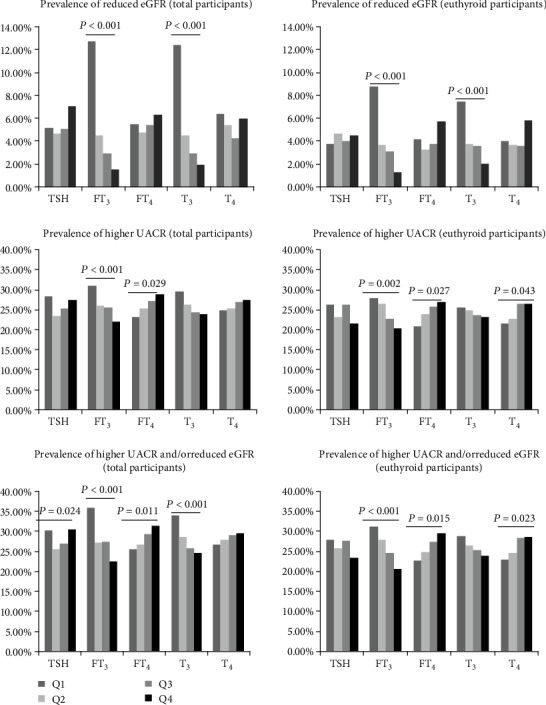
The prevalence of reduced eGFR, higher UACR, and higher UACR and/or reduced eGFR according to thyroid hormone quartile levels.

Then, we analyzed the prevalence of reduced eGFR, higher UACR, and higher UACR and/or reduced eGFR according to the quartile levels of thyroid hormones (Figure 2). In all participants, we observed that the prevalence of reduced eGFR decreased with increasing levels of FT_3_ and T_3_ quartiles (both *P* < 0.001). In terms of the prevalence of higher UACR and higher UACR and/or reduced eGFR, we found that they were negatively associated with quartiles of FT_3_ or T_3_ (both *P* < 0.001) and were positively associated with quartiles of FT_4_ (*P* = 0.029 and 0.011, respectively). Although there was a significant difference between the prevalence of higher UACR and/or reduced eGFR and TSH levels, it was not linearly correlated but was instead a “U shape”; that is, the lowest and highest quartiles of TSH have a higher prevalence of higher UACR and/or reduced eGFR. We obtained similar results when we grouped the total participants according to TSH reference ranges (<0.27 mIU/L, 0.27~4.20 mIU/L, and >4.2 mIU/L). The prevalence of reduced eGFR was 6.7%, 5.0%, and 8.0% (*P* = 0.008); the prevalence of higher UACR was 53.3%, 25.0%, and 31.0% (*P* < 0.001); and the prevalence of higher UACR and/or reduced eGFR was 53.3%, 26.8%, and 34.4% (*P* < 0.001), respectively.

### 3.3. Association of Thyroid Parameters with UACR and eGFR

To more effectively reveal the relationship of thyroid parameters with two main important indicators (UACR and eGFR) of diabetic kidney status, linear regression analysis was used.  Table 2 summarizes the results of the models. UACR and eGFR were both ln-transformed because of their skewed distribution. After adjusting for age, sex, the duration of diabetes, current smoking, HbA1c, UA, BMI, dyslipidemia, hypertension, and current use of metformin, UACR levels were negatively associated with the level of FT_3_ (*B* -0.171; 95% CI -0.242, -0.099; and *P* < 0.001) and T_3_ (*B* -0.269; 95% CI -0.408, -0.130; and *P* < 0.001). In addition, in all participants, eGFR was positively associated with the level of FT_3_ (*B* 0.069; 95% CI 0.0.057, 0.080; and *P* < 0.001) and T_3_ (*B* 0.096; 95% CI 0.074, 0.118; and *P* < 0.001) and was negatively associated with the level of TSH (*B* -0.007; 95% CI -0.010, -0.004; and *P* < 0.001), FT_4_ (*B* -0.003; 95% CI -0.006, 0.000; and *P* = 0.031), and TgAb positivity (*B* -0.029; 95% CI -0.056, -0.003; and *P* = 0.028). Moreover, there was no other significant association of UACR and eGFR with thyroid parameters (*P* > 0.05).

### 3.4. Association of Thyroid Hormones and Antibodies with the Prevalence of Kidney Disorders

**Figure 3:**
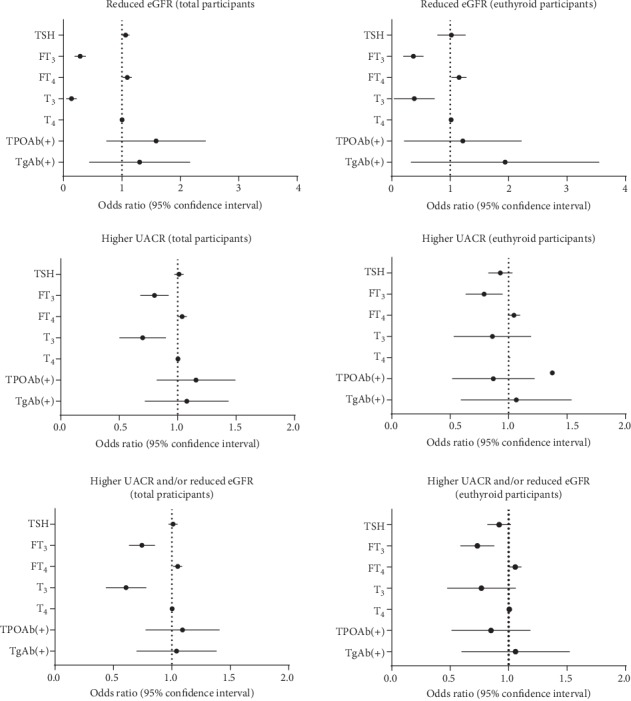
Associations of thyroid hormones and antibodies with reduced eGFR, higher UACR, and higher UACR and/or reduced eGFR. They were analyzed by using logistic regression. The regression models were adjusted for age, sex, HbA1c, BMI, UA, the duration of diabetes, current smoking, dyslipidemia, hypertension, and current use of metformin.

Given that thyroid status was associated with kidney disease risk factors (UACR and eGFR), we evaluated the adjusted odds ratios (ORs) for the prevalence of kidney disorder status (reduced eGFR, higher UACR, and higher UACR and/or reduced eGFR). Adjusted ORs were calculated after adjusting for age, sex, the duration of diabetes, current smoking, HbA1c, UA, BMI, dyslipidemia, hypertension, and current use of metformin using binary logistic regression models. As shown in Figure 3, higher TSH (OR 1.062; 95% CI 1.004, 1.124; and *P* = 0.036) and FT_4_ (OR 1.090; 95% CI 1.020, 1.164; and *P* = 0.011) and lower FT_3_ (OR 0.276; 95% CI 0.200, 0.380; and *P* < 0.001) and T_3_ (OR 0.119; 95% CI 0.063, 0.226; and *P* < 0.001) were associated with higher prevalence of reduced eGFR. Higher FT_4_ (OR 1.038; 95% CI 1.003, 1.075; and *P* = 0.033) and lower FT_3_ (OR 0.796; 95% CI 0.687, 0.922; and *P* = 0.002) and T_3_ (OR 0.682; 95% CI 0.514, 0.906; and *P* = 0.008) were associated with a higher prevalence of higher UACR. Similarly, higher FT_4_ (OR 1.050; 95% CI 1.015, 1.086; and *P* = 0.005) and lower FT_3_ (OR 0.739; 95% CI 0.639, 0.855; and *P* < 0.001) and T_3_ (OR 0.593; 95% CI 0.447, 0.785; and *P* < 0.001) were associated with a higher prevalence of higher UACR and/or reduced eGFR. No thyroid antibodies were associated with kidney disorders.

We then compared the AUROC for reduced eGFR, higher UACR, and higher UACR and/or reduced eGFR between TSH, FT_3_, T_3_, FT_4_, and T_4_. We found that the AUROC for FT_3_ was 0.742 for reduced eGFR, 0.552 for higher UACR, and 0.571 for higher UACR and/or reduced eGFR, which was higher than other thyroid parameters (Table 3). Considering the efficiency of the AUROC (>0.7), we calculated only the cutoff values of FT_3_ and T_3_ for reduced eGFR; they were 4.38 pmol/L and 1.49 nmol/L, respectively.

### 3.5. Association of Thyroid Homeostasis Parameters with Kidney Disorders

To reveal the relationship between thyroid homeostasis parameters and kidney disorders, we further evaluated the adjusted odds ratios (ORs) of SPINA-G_D_, SPINA-G_T_, TSHI, and TTSI for the prevalence of kidney disorders. Adjusted ORs were calculated. As shown in Table 4, lower SPINA-G_D_ was significantly associated with these three kidney disorder statuses, and higher TSHI and TTSI were also significantly associated with reduced eGFR (all *P* < 0.05).

### 3.6. Sensitivity Analyses

Further, we wanted to explore the relationship between thyroid parameters and reduced kidney function in participants with normal thyroid function and without euthyroid sick syndrome (ESS). Similar results were found for all participants.

Among these euthyroid participants, the prevalence of reduced eGFR, higher UACR, and higher UACR and/or reduced eGFR increased significantly along with decreasing levels of FT_3_ and/or T_3_. We also found that the prevalence of higher UACR and higher UACR and/or reduced eGFR increased with the levels of FT_4_ and T_4_ (Figure 2). UACR levels were negatively associated with the level of FT_3_, while eGFR was positively associated with the level of FT_3_ and T_3_ but was negatively associated with the level of FT_4_, T_4_, and TgAb positivity (Table 2). In logistic analysis, we also found lower FT_3_ or T_3_ levels, but higher FT_4_ or T_4_ levels were associated with the increased risks of reduced eGFR and higher UACR and/or reduced eGFR. Higher UACR was only negatively associated with FT_3_ (Figure 3). The AUROC of FT_3_ was 0.698 for reduced eGFR, 0.548 for higher UACR, and 0.562 for higher UACR and/or reduced eGFR, which was higher than other thyroid parameters (Table 3). Regarding the association of thyroid homeostasis parameters with kidney disorders in euthyroid participants, it was found that only lower SPINA-G_D_ was significantly associated with these three kidney disorder statuses (Table 4).

## 4. Discussion

Diabetic nephropathy, as one of the complications of diabetes, is related to the health and longevity of diabetic patients. The gold standard for the diagnosis of DKD is through kidney biopsy, which is difficult to achieve in a large population of samples. Several studies have suggested that morning UACR, as an important risk predictor for diabetic nephropathy and renal events that may provide prognostic information [26, 27], is an easy way to obtain the same information as a 24 h urine collection, which has been considered the gold standard [26, 28]. Thus, in our study, we focused on two main indictors of CKD and DKD [29], reduced eGFR and higher UACR.

Metformin, as a first-line treatment for type 2 diabetes, was reported to have some effect on the hypothalamic-pituitary-thyroid axis [30], and it was also associated with a decrease in the levels of TSH, possibly by enhancing the effects of thyroid hormones in the pituitary and activating adenosine monophosphate-activated protein kinase (AMPK) [31]. Thus, in our study, the current use of metformin was taken into account in all regression analyses.

In our study, among all participants, we found that the lowest and highest quartile levels of TSH had a higher prevalence of reduced eGFR, higher UACR, and higher UACR and/or reduced eGFR. Previous studies reported that both hypothyroidism and hyperthyroidism worsen kidney function directly by affecting renal blood flow, GFR, tubular function, electrolyte homeostasis, electrolyte pump functions, and kidney structure (e.g., decreased glomerular volume and area) and increasing peripheral vascular resistance [14–16], leading to higher serum CR concentrations and a lower eGFR. Several researchers also found an association of subclinical hypothyroidism with renal injury, such as macroalbuminuria in DM subjects [32–35]. Similarly, in our current study, the prevalence of these three kidney disorders was significantly different when all participants were grouped by the reference range of TSH, the distribution of which looked like a “U” shape. Thus, we hypothesized that thyroid dysfunction, including overt and subclinical hypothyroidism or hyperthyroidism, was associated with kidney disorders in DM participants. On the other hand, it is worth pointing out that this association failed to be found when it was analyzed among participants with normal thyroid function in this study. Conflicting evidence has been reported about the association of TSH and kidney status in euthyroid DM subjects [14, 36, 37]. The HUNT study reported that TSH within the reference range was negatively associated with eGFR [14] in type 2 diabetes, while others [7, 38] reached a different conclusion.

In terms of other thyroid hormones, we observed increased prevalence of reduced eGFR, higher UACR, and higher UACR and/or reduced eGFR along with decreased levels of FT_3_ and T_3_. After full adjustment, FT_3_ and T_3_ levels were significantly negatively associated with UACR and were positively associated with eGFR. In logistic analysis, lower FT_3_ and T_3_ were also found to be associated with increased risks of reduced eGFR, higher UACR, and higher UACR and/or reduced eGFR. Furthermore, though we found that the AUROC of FT_3_ for these three patterns of kidney disorders was much higher than that for any other thyroid hormones, only the AUROC of FT_3_ and T_3_ for reduced eGFR in all participants was larger than 0.7, which may give some significant values. The cutoff values of lower FT_3_ and T_3_ for reduced eGFR were 4.38 pmol/L and 1.49 nmol/L, respectively. These results were in agreement with some other cohort and cross-sectional studies that suggested that lower T_3_ has been consistently found to be the most common disturbance in patients with kidney disorders and that it was an independent predictor of survival in various illness states [12, 36, 39–42]. However, only a few studies have focused on the relationship between normal thyroid hormone levels and renal events in DM participants. In our present study, these results were strongly consistent even though FT_3_ and T_3_ levels were in the normal reference range. In addition, FT_3_ but not FT_4_ was reduced in kidney disorders, suggesting that low FT_3_ is unlikely to be an in vitro artifact because substances inhibiting binding are expected to affect FT_4_ to a greater extent than FT_3_. Thus, reduced FT_3_ seems to reflect a true selective T_3_ deficiency due to the inhibition of 5′-deiodinase, which is a catalyzing enzyme for the production of T_3_ from circulating T_4_ [39, 43]. Higher FT_4_ and lower T_3_ concentrations were reported to be strongly and independently associated with renal events [44]. In this study, we obtained the same results.

It has been suggested that autoimmune thyroid disease may lead to the deposition of immunocomplexes in the renal glomeruli and may be responsible for endothelial dysfunction and subsequent microalbuminuria [45]. In the present study, both among all participants and among euthyroid participants, TgAb positivity was significantly associated with reduced eGFR, which suggested that thyroid autoimmunity may be involved in renal injury. However, there are different points of view. Suher et al. reported that the association with eGFR was roughly similar for hypothyroidism with and without TPOAb positivity, which suggested that hypothyroidism-associated kidney dysfunction seems to be more related to the decline in thyroid hormone levels than to thyroid autoimmunity [46].

Finally, we explored the relationship of thyroid homeostasis parameters, including two structural parameters of thyroid homeostasis (SPINA-G_D_ and SPINA-G_T_) and two pituitary thyrotropic function indices (TSHI and TTSI), with kidney status. By extending the classic concept of separate measurements of thyroid hormone parameters, these markers add new qualitative and quantitative dimensions to the evaluation of thyroid homeostasis, offer a more integrated and systemic view, and deliver important insights into the physiology of pituitary-thyroid feedback control [20]. Though it was reported that SPINA-G_T_ was correlated with creatinine clearance [20, 21], we found that lower SPINA-G_D_ was significantly associated with three kidney disorder statuses, and higher TSHI and TTSI were significantly associated with reduced eGFR. Therefore, it was suggested that kidney disorders may be related to the maximum stimulated activity of step-up deiodination and pituitary thyrotropic function.

Though our study had some strengths, including novelty, the analysis of community dwelling participants with a relatively large sample size, and strong quality control, there were also some limitations. First, because of the cross-sectional study, we can evaluate association but not causality. We conducted a Mendelian randomization (MR) analysis to examine the causality of the association between TSH and eGFR in 10603 participants recruited from the Survey on Prevalence in East China for Metabolic Diseases and Risk Factors (SPECT-China, ChiCTR-ECS-14005052, http://www.chictr.org.cn) [25, 47]. By a genetic approach that limits residual confounding and reverse causation in observational conventional epidemiological studies, TSH and eGFR were not causally associated, which suggested that genetically elevated TSH concentrations may not affect renal function [48]. The mechanism linking kidney function and thyroid homeostasis remains unclear. Thyroid dysfunction may trigger kidney failure, and renal failure might trigger an allostatic response of thyroid function as well, i.e., euthyroid sick syndrome [49] or thyroid allostasis in critical illness, tumors, uremia, and starvation (TACITUS) [50], albeit in slight form. This is known from previous studies and is plausible because the kidney expresses deiodinases that catalyze the conversion of T_4_ to T_3_. Further prospective and longitudinal studies are needed. Second, UACR and thyroid function were estimated only once, which could result in misleading classifications. Third, the levels of rT_3_ were not measured in this study.

## 5. Conclusion

Among type 2 diabetes patients, elevated TSH and FT_4_ (or T_4_), lower FT_3_ (or T_3_), and TgAb positivity were associated with higher UACR and lower eGFR levels. Elevated TSH and FT_4_ (or T_4_), lower FT_3_ (or T_3_) levels, and thyroid homeostasis parameters (SPINA-G_D_, TSHI, and TTSI) were associated with the prevalence of kidney disorders. The lower FT_3_, even within a normal reference range, was the factor most related to reduced eGFR compared with all other thyroid hormones. Multidisciplinary collaborative care, including nephrologists, endocrinologists, or internal medicine specialists, is needed and appears to positively impact the clinical, humanistic, and economic outcomes of patients with diabetes [51, 52].

## Figures and Tables

**Table 1:** Clinical characteristics of all participants.

	All	Men	Women
*N*	4136	1983	2153
Age (yr)	67.23 ± 8.63	67.50 ± 8.66	66.97 ± 8.61
Duration of diabetes (yr)	9.00 (12.00)	10.00 (12.00)	8.00 (12.00)
Current smoking (%)	18.7	36.3	2.4
BMI (kg/m^2^)	24.97 ± 3.58	24.99 ± 3.29	24.96 ± 3.82
HbA1c (%)	7.20 (1.60)	7.30 (1.60)	7.10 (1.50)
UA (*μ*mol/L)	327.55 ± 81.41	343.79 ± 82.08	312.60 ± 77.87
Hypertension (%)	84.6	84.5	84.8
Dyslipidemia (%)	62.0	63.5	60.5
UACR (mg/g Cr)	13.00 (23.00)	13.00 (24.00)	14.00 (23.00)
eGFR (ml/min/1.73 m^2^)	95.63 (18.59)	94.56 (18.64)	96.66 (18.02)
Reduced eGFR (%)	5.58	6.0	5.1
Higher UACR (%)	26.0	25.9	26.2
Higher UACR and/or reduced eGFR (%)	28.1	27.8	28.4
Thyroid parameters			
TSH (mIU/L)	2.53 (1.78)	2.33 (1.58)	2.73 (1.97)
FT_3_ (pmol/L)	4.63 ± 0.60	4.78 ± 0.60	4.50 ± 0.57
FT_4_ (pmol/L)	16.76 ± 2.29	16.96 ± 2.30	16.58 ± 2.26
T_3_ (nmol/L)	1.65 ± 0.29	1.66 ± 0.30	1.64 ± 0.28
T_4_ (nmol/L)	101.05 ± 17.84	98.95 ± 17.99	102.99 ± 17.49
TPOAb positive (%)	7.7	5.2	10.0
TgAb positive (%)	6.2	3.3	9.0
Thyroid homeostasis parameters			
SPINA-G_D_ (nmol/s)	15.33 ± 3.12	15.25 ± 3.23	15.41 ± 3.02
SPINA-G_T_ (pmol/s)	2.31 (1.05)	2.38 (1.05)	2.25 (1.06)
TSHI	3.17 ± 0.58	3.13 ± 0.57	3.22 ± 0.59
TTSI (m/L)	192.32 (132.76)	179.96 (122.53)	207.08 (143.21)

Normally and nonnormally distributed continuous variables were expressed as mean ± SD and median (interquartile range), respectively. BMI: body mass index; HbA1c: glycated hemoglobin; UA: uric acid; UACR: urinary albumin to creatinine ratio; Cr: creatinine; eGFR: estimated glomerular filtration rate; TSH: thyroid-stimulating hormone; FT_3_: free triiodothyronine; FT_4_: free thyroxine; T_4_: thyroxin; T_3_: triiodothyronine; TPOAb: thyroid peroxidase antibody: TgAb: thyroglobulin antibody; SPINA-G_D_: sum activity of peripheral deiodinases; SPINA-G_T_: thyroid's secretory capacity; TSHI: Jostel's TSH index; TTSI: thyrotroph thyroid hormone resistance index.

**Table 2:** Association of thyroid parameters with UACR and eGFR by linear regression.

	lnUACR		lneGFR	
	*B* (95% CI)	*P*	*B* (95% CI)	*P*
Total participants				
TSH	0.014 (-0.004, 0.033)	0.136	-0.007 (-0.010, -0.004)	<0.001
FT_3_	-0.171 (-0.242, -0.099)	<0.001	0.069 (0.057, 0.080)	<0.001
FT_4_	0.008 (-0.010, 0.025)	0.399	-0.003 (-0.006, 0.000)	0.031
T_3_	-0.269 (-0.408, -0.130)	<0.001	0.096 (0.074, 0.118)	<0.001
T_4_	0.000 (-0.002, 0.002)	0.896	-0.000 (-0.000, 0.000)	0.752
TPOAb positive	0.092 (-0.054, 0.238)	0.215	-0.023 (-0.046, 0.000)	0.053
TgAb positive	-0.004 (-0.168, 0.160)	0.961	-0.029 (-0.056, -0.003)	0.028
Euthyroid participants				
TSH	-0.029 (-0.080, 0.021)	0.258	-0.004 (-0.011, 0.003)	0.304
FT_3_	-0.124 (-0.214, -0.033)	0.008	0.046 (0.034, 0.059)	<0.001
FT_4_	0.011 (-0.012, 0.033)	0.354	-0.005 (-0.008, -0.002)	0.001
T_3_	-0.088 (-0.269, 0.092)	0.337	0.045 (0.020, 0.070)	<0.001
T_4_	0.002 (-0.001, 0.004)	0.264	-0.001 (-0.001, 0.000)	0.002
TPOAb positive	-0.005 (-0.186, 0.177)	0.958	-0.004 (-0.029, 0.022)	0.782
TgAb positive	-0.024 (-0.231, 0.184)	0.822	-0.031 (-0.060, -0.002)	0.033

UACR and eGFR were ln-transformed for normal distribution before linear regression analysis. The regression models were adjusted for age, sex, HbA1c, BMI, urine acid, the duration of diabetes, current smoking, dyslipidemia, hypertension, and current use metformin.

**Table 3:** Comparison of AUROC between thyroid hormones for kidney disorders.

	Reduced eGFR	*P*	Higher UACR	*P*	Higher UACR and/or reduced eGFR	*P*
Total participants						
TSH	0.549	0.014	0.502	0.881	0.506	0.517
FT_3_	0.742	<0.001	0.552	<0.001	0.571	<0.001
FT_4_	0.521	0.276	0.528	0.007	0.531	0.002
T_3_	0.705	<0.001	0.535	0.001	0.551	<0.001
T_4_	0.486	0.479	0.515	0.130	0.515	0.123
Euthyroid participants						
TSH	0.518	0.490	0.486	0.241	0.486	0.242
FT_3_	0.698	<0.001	0.548	<0.001	0.562	<0.001
FT_4_	0.544	0.093	0.529	0.018	0.533	0.006
T_3_	0.645	<0.001	0.511	0.368	0.525	0.034
T_4_	0.542	0.104	0.535	0.004	0.536	0.002

Data are presented as the area under the receiver operating characteristic curves (AUROC).

**Table 4:** Associations of thyroid homeostasis parameters with kidney disorders.

	Reduced eGFR		Higher UACR		Higher UACR or reduced eGFR	
	OR (95% CI)	*P*	OR (95% CI)	*P*	OR (95% CI)	*P*
Total participants						
SPINA-G_D_	0.801 (0.754, 0.852)	<0.001	0.951 (0.926, 0.977)	<0.001	0.935 (0.910, 0.960)	<0.001
SPINA-G_T_	0.993 (0.937, 1.052)	0.814	1.013 (0.994, 1.032)	0.187	1.012 (0.994, 1.030)	0.197
TSHI	1.349 (1.021, 1.783)	0.035	1.027 (0.896, 1.178)	0.703	1.050 (0.917, 1.202)	0.479
TTSI	1.001 (1.000, 1.002)	0.018	1.000 (1.000, 1.001)	0.294	1.000 (1.000, 1.001)	0.276
Euthyroid participants						
SPINA-G_D_	0.831 (0.755, 0.913)	<0.001	0.961 (0.926, 0.996)	0.031	0.947 (0.914, 0.982)	0.003
SPINA-G_T_	1.054 (0.866, 1.283)	0.601	1.064 (0.973, 1.162)	0.173	1.065 (0.976, 1.162)	0.157
TSHI	1.449 (0.934, 2.248)	0.098	1.000 (0.821, 1.218)	0.999	1.017 (0.837, 1.235)	0.866
TTSI	1.000 (1.000, 1.000)	0.437	1.000 (1.000, 1.000)	0.538	1.000 (1.000, 1.000)	0.501

They were analyzed by using logistic regression. The regression models were adjusted for age, sex, HbA1c, BMI, UA, the duration of diabetes, current smoking, dyslipidemia, hypertension, and current use of metformin.

## Data Availability

The data used to support the findings of this study are included within the article.

## Abbreviations

DM: Diabetes mellitus
CKD: Chronic kidney disease
DKD: Diabetic kidney disease
TH: Thyroid hormone
UACR: Urinary albumin to creatinine ratio
CR: Creatinine
eGFR: Estimated glomerular filtration rate
TSH: Thyroid-stimulating hormone
FT_3_: Free triiodothyronine
FT_4_: Free thyroxine
T_4_: Thyroxin
T_3_: Triiodothyronine
TPOAb: Thyroid peroxidase antibody
TgAb: Thyroglobulin antibody
BMI: Body mass index
HbA1c: Glycated hemoglobin
CAP: College of American Pathologists
CKD-EPI: Chronic Kidney Disease Epidemiology Collaboration
TG: Triglycerides
TC: Total cholesterol
HDL-C: High-density lipoprotein
LDL-C: Low-density lipoprotein
UA: Uric acid
AUROC: Area under the receiver operating characteristic curve
ORs: Odds ratios
SPINA-G_D_: Sum activity of peripheral deiodinases
SPINA-G_T_: Thyroid's secretory capacity
TSHI: Jostel's TSH index
TTSI: Thyrotroph thyroid hormone resistance index.
